# Mental health, quality of life and mental health service use after the COVID-19 pandemic: Results from a cross-sectional nationwide study among German children and adolescents

**DOI:** 10.1186/s12889-026-28159-6

**Published:** 2026-06-19

**Authors:** Hannah Elisabeth Köhler, Anna-Lina Rauschenbach, Kristin Rodney-Wolf, Judith Bauch, Franziska Greiner-Döchert, Henrik Saalbach, Eva Baumann, Julian Schmitz

**Affiliations:** 1 Department of Clinical Child and Adolescent Psychology, Leipzig University, Leipzig, Germany; 2 Department of Educational Psychology, Leipzig University, Leipzig, Germany; 3 Department of Journalism and Communication Research, Hanover University of Music, Drama and Media, Hanover, Germany

**Keywords:** Mental health, Quality of life, Help-seeking, Children and adolescents

## Abstract

**Background:**

The COVID-19 pandemic significantly impacted the mental health of children and adolescents, particularly those from low-income families and adolescent girls. The long-term effects remain unclear. This study assessed the mental health measured by emotional and behavioural problems and quality of life (QoL) among German children and adolescents, emphasising socio-economic status and gender differences. The help-seeking behaviour among families of children with mental health problems was also examined.

**Methods:**

A nationwide representative online survey was conducted in April/May 2024. *N* = 1,530 children and adolescents (ages 8–17) and their parents participated. The self- and parent-report survey assessed behavioural and emotional problems (Strength and Difficulties Questionnaire). QoL (KIDSCREEN-10 Index) was rated in self-report. Parents reported on past help-seeking behaviour within and outside of school via items constructed for the present study.

**Results:**

Behavioural and emotional problems (21.1% in self-report, 22.4% in parental report) and low QoL (27.3%) were higher than pre-pandemic levels. Children from low-income families were particularly affected. Of all parents, 23.7% reported a need for help due to their child’s mental health issues in the last 12 months, with 28.4% of these parents not seeking professional support. Among those who sought help, most contacted multiple services, with teachers, psychotherapists and general practitioners being the most frequently contacted services. Families who secured a therapy placement waited an average of 12 weeks for an initial appointment and 18 weeks to begin therapy.

**Conclusions:**

Behavioural and emotional problems as well as low QoL seem to stabilise at a rate higher than the pre-pandemic baseline, indicating a need for ongoing monitoring, adjustment to the mental health system and further research on correlates and causes of this stabilisation.

**Supplementary Information:**

The online version contains supplementary material available at 10.1186/s12889-026-28159-6.

## Background

Childhood and adolescence are sensitive periods, as most mental disorders emerge during this time [1, 2]. The onset of mental disorders in these stages can significantly disrupt further development and, if left untreated, these disorders may become chronic and lead to comorbidities and secondary conditions [1, 2]. During the COVID-19 pandemic, the mental health of children and adolescents eroded worldwide [3, 4]. In Germany, between 23% and 31% of school-aged children exhibited behavioural and emotional problems during the pandemic, representing a substantial increase from the pre-pandemic rate of 17% [5, 6]. Beside a rise in mental health problems, quality of life (QoL) of children and adolescents, a subjective evaluation of one’s physical, psychological and social well-being in one’s specific environment, was also negatively affected [7]. QoL has increasingly been recognised as a critical health outcome, particularly for assessing the impact of illness on an individual’s overall well-being [8, 9]. Although QoL and mental health are correlated, they represent distinct constructs, each contributing unique insights [10, 11]. Consequently, both domains were assessed in this study to provide a more holistic understanding of youth well-being. Between 27% and 48% of children and adolescents reported low QoL during the pandemic, a significant increase from the pre-pandemic rate of 15% [5, 12].The trajectory of mental health issues and QoL in the post-pandemic period remains uncertain, as pre-pandemic prevalence rates have not been restored as of the end of 2022 [5, 13]. Furthermore, new crises emerge, impacting the mental health of young people in Germany [14, 15]. Mapping post-pandemic trends is crucial, as treatment capacities must be adjusted to prevent negative long-term effects.

Not all children and adolescents were affected equally by the pandemic. Particularly children and adolescents in high-risk groups are vulnerable to negative consequences of prolonged stressors, such as a global pandemic, and other global crises, such as climate change. A substantial body of research indicates that low socioeconomic status (SES) is associated with an increased risk of mental illness and a lower quality of life [16, 17]. Children and adolescents with low SES are exposed to multiple stressors, including stressful life events, while simultaneously possessing fewer resources [17, 18]. Some evidence suggests that children and adolescents from low-SES families experienced a particularly strong increase in mental health issues during the pandemic [4, 19]. Gender-based disparities have also emerged. Female adolescents appear to be more vulnerable to adverse psychological responses, such as post-traumatic stress disorder, following stressful life events [20]. Moreover, this subgroup demonstrates a greater propensity for internalising disorders and is particularly affected by their increase in recent years [21, 22]. Consistent with these observations, administrative data from German statutory health insurance (SHI) providers and the Central Institute for SHI Physicians has documented a significant increase in depressive, anxiety and eating disorders among female adolescents during the pandemic [23]. Given these considerations, a closer examination of the prevalence of mental health issues in these predefined risk subgroups is needed. To achieve this goal, we performed subgroup analyses within the framework of this population-based study.

To estimate the prevalence of mental health issues in children and adolescents, many studies rely on parent-proxy reports [5]. However, such reports are susceptible to bias from parents’ own emotional state and health [24, 25]. In contrast, youth self-reports tend to provide more accurate measurements particularly for internalising symptoms [24, 26, 27], which have increased during the pandemic [3, 4]. Youth self-reports have consistently demonstrated reliability as a data source [26, 28]. Consequently, the present study prioritises self-reports from children and adolescents wherever feasible, foregrounding this critical perspective.

Bearing in mind the elevated prevalence of mental health issues and reduced quality of life in German children and adolescents during the pandemic, access to psychological treatment has become increasingly important. Psychotherapy financed by state-funded health insurance in Germany encompasses psychodynamic, cognitive-behavioural and systemic therapy [29]. Prior to the COVID-19 pandemic, 5.9% of German children and adolescents utilised mental health services on a temporary basis [30]. During the pandemic, therapists reported a surge in demand for therapy, leading to longer waiting times [23]. Additionally, there was an increase in referrals for psychiatric assessment and hospitalisation for children and adolescents [31]. These trends prompted calls for the expansion of existing mental health care structures [31, 32]. Low-threshold support structures are equally important, as they often serve as a first point of contact for initiating treatment. Schools play a critical role in this regard, because they are where children spend a significant amount of time and teachers are often the first to recognise and respond to emerging mental health concerns [33]. However, there is little data on how low-threshold support structures are accessed. Reliable data on the demand for and utilisation of different support services is crucial for better planning and adjustment of existing structures. Therefore, the present study examines perceived need for support, help-seeking behaviour, service access and psychotherapy waiting times, integrating findings with current prevalence estimates to advance the research on this topic.

Taking the results and limitations of previous studies as a starting point, the current study aimed to answer the following questions:

1. What is the current prevalence of behavioural and emotional problems in children and adolescents (ages 8–17) in Germany? Which of the predefined risk subgroups exhibit higher rates of behavioural and emotional problems?
2. What proportion of children and adolescents in Germany meet the criteria for low QoL? Do a higher proportion of children and adolescents from the predefined risk subgroups meet the criteria for low QoL compared to their peers?
3. How many parents report a need for support due to their child’s mental health problems? How many seek help and where? How do parents assess the helpfulness of the support they receive?
4. How accessible is psychotherapy for families, as it is the gold standard of care for mental health issues in children and adolescents?

## Methods

### Study design and sample

Data was collected in the nationwide, population-based study “German School Barometer”, conducted online from 26 April to 20 May 2024 and approved by the local ethics committee. Participants were selected from the forsa.omninet online panel, a representative sample of over 150,000 German-speaking participants. For this study, panel members between the ages of 25 to 65 were randomly drawn and screened as to whether they had a child in the appropriate age range (8–17 years) who attended school. If parents had several children, the child who participated in the study was chosen randomly by selecting the child with the most recent birthday. Informed consent was obtained from participants. The study included *N* = 1,530 families. Children between the ages of 8 and 10 (*n* = 405) were presented with a shortened version of the questionnaire since some measures were not designed for this age range. Parental reports were gathered from one parent of each family (*n* = 1,530). Weighted data of the study sample matched the sociodemographic characteristics of the German population (based on data by the Federal Statistical Office of Germany). The individual weights were calculated based on gender, age and education of the parents; gender, age and school type of the child; and region. They ranged from 0.2 to 7.4. A power analysis showed a minimum sample size of *n* = 31 participants per group for detecting a medium effect (*f* = 0.3) with a significance level of *p* < .05 and a power of *p* (1-β) = 0.8 between two groups of a particular age (8–13 years, 14–17 years) and gender (female vs. male).

### Measures

#### Sociodemographic variables

For sociodemographic information, the parental survey included questions on their child’s age, gender, school grade, type of school and migration background. Income was measured via the equivalised disposable income. Low income was defined as less than 70% and high income as more than 150% of the median equivalised disposable income in Germany in 2023 [34]. Education was categorised using the CASMIN classification (comparative analysis of social mobility in industrial nations) [35, 36]. The parental education level was calculated by assigning the higher score of both parents as the total score. Child age and gender along with family income and parental education level were employed as stratification variables in subgroup analyses to delineate predefined risk subgroups.

#### Behavioural and emotional problems

The Strength and Difficulties Questionnaire (SDQ) was used to assess behavioural and emotional problems [37]. It was administered to all parents and adolescents aged 11 to 17 years. The SDQ consists of 20 items which can be aggregated into a sum score as well as four problem scales with five items each: “emotional symptoms”, “conduct problems”, “hyperactivity” and “peer problems”. Response options ranged from 0 (“not true”) to 2 (“certainly true”). We categorised participants’ sum scores into “abnormal”, “borderline” or “normal” groups based on German norms, with gender-neutral and ageless norms used for diverse genders [38, 39].

Internal consistency in this study was good (Cronbach’s *α* for children’s version = 0.81, parental report = 0.83). The internal consistencies of the subscales varied (Cronbach’s *α* = 0.43–0.81) but were mostly sufficient. Only the subscale “conduct problems” had insufficient internal consistencies (Cronbach’s *α* for children’s version = 0.43, parental report = 0.57). Given previous reports of moderate to low internal consistencies for the subscales, the data should be interpreted cautiously [38, 40].

#### Quality of life

To measure QoL, the KIDSCREEN-10 Index was presented to all children and adolescents aged 8–17 for self-report. The KIDSCREEN-10 consists of ten items, covering the physical, psychological, mental and social aspects of QoL [41]. Participants were presented with a 5-point response scale (0 = “not at all” to 4 = “extremely” or 0 = “never” to 4 = “always”). Sum scores were categorised into “low”, “medium” and “high” QoL according to age- and gender-specific national cut-offs [41]. For respondents with a diverse gender, general cut-offs were used. The scale showed good internal consistency in this study (Cronbach’s *α* = 0.83). As the term “health-related quality of life” remains contested and the KIDSCREEN-10 primarily assesses overall quality of life, we adopt the term “quality of life (QoL)”, consistent with other studies in the field [42, 43].

#### Help-seeking behaviour

To explore help-seeking behaviour, the parental report included items which were developed for this study by a group of experts based on typical structures of mental health services in Germany (for the survey, please see Additional File 1). The items assessed parents’ perceived need for support for their child and if they sought support in the last twelve months in a yes/no format. If parents had sought help, they were asked which services they accessed. School-based services (e.g. school psychologist, guidance counsellor) and non-school based services (e.g. general practitioner, psychotherapist) were included. Parents rated the helpfulness of accessed services on a four-point Likert-scale (1 = “not helpful at all” to 4 = “very helpful”). If families tried to access psychotherapy, they were asked for the number of practices contacted, waiting time for an initial appointment and, where applicable, the start of therapy in an open format.

### Data analysis

Categories for SDQ and KIDSCREEN-10 were created using national norm data and presented as descriptive statistics [38, 39, 41]. Differences by age, gender, education and income were evaluated via chi-square tests and analyses of variance (ANOVAs). Age groups were defined as 8–13 years for children/pre-adolescents and 14–17 years for adolescents. Chi-square tests were used for SDQ/KIDSCREEN-10 categorisation with effect sizes calculated as Cramer’s *V* (.10 indicating a small, .30 a medium and .50 a strong effect). ANOVAs were used for sum-score analysis, with eta squared as effect size and Tukey’s honest significance test (Tukey’s HSD) as a post-hoc test.

Questions on help-seeking behaviour and access to psychotherapy were reported descriptively at item level. To test differences in user frequencies of services, a chi-square goodness of fit test was calculated. Since frequencies are not independent in this sample, calculations were exploratory. To test the influence of the SDQ score on the likelihood of starting therapy, a logistic regression was calculated. For all analyses, data weighted according to sociodemographic characteristics of the German population was used. Missing data was sporadic and complete case analysis was used.

## Results

### Sample characteristics

Data from *N* = 1,530 students aged 8–17 years (48.8% female, *M*_age_ = 12.54 years, *SD*_age_ = 2.60) and one of their parents was analysed. Most families were classified as middle class (63.6%) with moderate parental education (53.7%). A migration background was reported among 12.0% of all children and adolescents.

Further details on the sample characteristics are given in Table 1.

**Table 1:** Sociodemographic characteristics of the sample

Characteristics		*n* (%)
Gender of the child/adolescent	Male	779 (50.9)
	Female	746 (48.8)
	Diverse	5 (0.3)
Migration background	Yes	184 (12.0)
	No	1346 (88.0)
Household income	Low	199 (13.0)
	Moderate	973 (63.6)
	High	165 (10.8)
	No information	193 (12.6)
Parental education (combination score)	Low	46 (3.0)
	Moderate	821 (53.7)
	High	663 (43.3)

Unweighted data

### Children and adolescents’ report

#### Behavioural and emotional problems

Self-reports from children and adolescents aged 11–17 years (*n* = 1,228) revealed 12.5% (*n* = 154) abnormal and 8.6% (*n* = 105) borderline SDQ scores. On a subscale level, emotional symptoms were classified as borderline or abnormal in 22.9% of the sample (*n* = 281), conduct problems in 10.4% (*n* = 128), hyperactivity in 14.2% (*n* = 174) and peer problems in 29.2% (*n* = 359).

ANOVA showed significant differences in SDQ scores between income groups (*F* (2, 972) = 3.18, *p* = .042, η² = .007), with the low-income group scoring higher than the high-income group (*p* = .037, 95% *C.I.* = 0.08, 3.28). Parental education had no significant effect (*p* = .112). A two-way ANOVA for age (11–13 years vs. 14–17 years) and gender (male vs. female) revealed a significant interaction (*F* (1, 1115) = 8.56, *p* = .004, η² = .008). Post-hoc tests showed adolescents boys had significantly lower SDQ scores than adolescent girls (*p* < .001, 95% *C.I.* = -3.13, -0.84), pre-adolescent girls (*p* = .007, 95% *C.I.* = -2.64, -0.30) and pre-adolescent boys (*p* = .014, 95% *C.I.* = -2.53, -0.20).

#### Quality of life

According to self-report, 27.3% (*n* = 418) of children and adolescents had low, 66.4% (*n* = 1,017) medium and 6.3% (*n* = 96) high KIDSCREEN-10 scores. KIDSCREEN-10 categories differed significantly by SDQ groups (reported by children and adolescents; *V* = .33, *p* < .001), with 73% of the 154 respondents with abnormal and 53% of the 105 respondents with borderline SDQ scores showing low KIDSCREEN-10 scores.

ANOVA revealed a significant effect of parental income on KIDSCREEN-10 scores (*F* (2, 1334) = 6.98, *p* = .001, η² = .010), with low-income children reporting significantly lower scores than medium-income children (*p* = .002, 95% *C.I.* = -2.42, -0.47). Parental education had no significant effect (*p* = .091).

A two-way ANOVA for age (8–13 vs. 14–17 years) and gender (male vs. female) showed that adolescent girls reported significantly lower scores (*F* (1, 1521) = 22.17, *p* < .001, η² = .014) than pre-adolescent boys (*p* < .001, 95% *C.I.* -3.24, -1.21), pre-adolescent girls (*p* < .001, 95% *C.I.* -3.52, -1.48) and adolescent boys (*p* < .001, 95% *C.I.* -3.42, -1.18).

### Parental report

#### Behavioural and emotional problems

In parental report, 12.9% (*n* = 197) of children and adolescents had abnormal and 9.5% (*n* = 146) borderline SDQ scores. On a subscale level, emotional symptoms were classified as borderline or abnormal in 27.4% (*n* = 419), conduct problems in 9.5% (*n* = 145), hyperactivity in 19.8% (*n* = 303) and peer problems in 25.4% (*n* = 388) of the sample^1^.

ANOVA showed significant differences in SDQ scores between income groups (*F* (2, 1334) = 6.41, *p* = .002, η² = .010), with higher scores in the low-income group compared to the medium- (*p* = .006, 95% *C.I.* = 0.32, 2.38) and high-income group (*p* = .011, 95% *C.I.* = 0.32, 3.10). Parental education also had a significant effect (*F* (2, 1527) = 7.15, *p* = .001, η² = .009), with the medium-education group scoring higher than the high-education group (*p* = .002, 95% *C.I.* = 0.33, 1.70).

A two-way ANOVA for age (8–13 vs. 14–17 years) and gender (male vs. female) showed a significant interaction effect (*F* (1, 1521) = 14.70, *p* < .001, η² = .009). The post-hoc test revealed that pre-adolescent boys had higher scores than pre-adolescent girls (*p* < .001, 95% *C.I.* 0.67, 2.55), adolescent boys (*p* < .001, 95% *C.I.* 1.29, 3.40) and adolescent girls (*p* < .001, 95% *C.I.* 0.68, 2.83).

#### Help-seeking behaviour

When asked whether their child needed help because of psychological issues in the last twelve months, 23.7% of all parents answered yes, 72.3% felt no help was needed and 4.0% did not comment.

Parents of children with abnormal (64%) and borderline (36%) SDQ scores were significantly more likely to report a need for help (*V* = .39, *p* < .001), as were those of children with low KIDSCREEN-10 scores (44%, *V* = .30, *p* < .001).

Of the parents reporting a need for help (*n* = 363), 70.3% sought help, 28.4% did not and 1.4% did not comment. There were no significant differences in help-seeking based on income (*p* = .658), gender (*p* = .197), age (*p* = .086) or (in exploratory analysis) parental educational (*p* = .845).

Parents that sought help (*n* = 255) mostly contacted both school and non-school based services (50.8%). A minority of 14.1% sought help only in school and 34.4% only outside school. Chi-square tests showed differences in help-seeking by age of the child (*V* = .28, *p* < .001). Parents of children up to 13 years were more likely to seek help from school services (19%) or both school and non-school services (59%), while parents of adolescents mainly sought help outside school (49%) and rarely exclusively within school (9%). Help-seeking behaviour did not differ depending on gender of the student (*p* = .157) or (in exploratory analysis) on income (*p* = .070) or parental education (*p* = .084).

The number of families seeking help with different services differed significantly in an exploratory test of equal proportions (*χ*^*2*^ (12) = 307.46; *p* < .001).

Within the school system, most families contacted their child’s class teacher (45.7%), followed by school social workers (25.1%) and school psychologists (20.0%). Outside the school system most families contacted psychotherapists (42.2%) or sought medical support through a general practitioner (32.6%) or a psychiatrist (27.1%). All data is presented in Table 2.

**Table 2:** Percentage of families who sought help with different services

	Service	Percentage	*n*
*Within school*	Any service	64.8	166
	Class teacher	45.7	117
	School social worker	25.1	64
	School psychologist	20.0	51
	Principal	13.3	34
	Guidance counsellor	11.7	30
	Another teacher	9.4	24
	Someone else	5.1	13
*Outside school*	Any service	85.1	217
	Psychotherapist	42.2	108
	General practitioner	32.6	83
	Psychiatrist	27.1	69
	Information centre	22.3	57
	Youth welfare office	21.2	54
	Somewhere else	5.1	13

Percentages relative to families who sought help in any form (*n* = 255)

Most parents who sought help reported receiving it (90.6%, *n* = 231), although not always from the service they initially sought it from. Specifically, 23.5% of parents (*n* = 39) seeking help at school and 12.9% (*n* = 28) outside school received no support in these contexts.

The services accessed were mostly rated as helpful (*M* = 3.13 to 3.54 with a maximum of 4.0, *n* = 17–92). The highest mean helpfulness ratings were received by principals and guidance counsellors (both *M* = 3.54). The lowest mean helpfulness ratings were given to school psychologists and general practitioners (both *M* = 3.13). Further information is given in Table 3.

**Table 3:** Helpfulness of services within and outside schools rated by parents as mean (*n*) and percentages

	Service	Mean rating (*n*)	Percentage “Very helpful”	Percentage “Somewhat helpful”	Percentage “Not very helpful”	Percentage ”Not helpful at all”
*Within school*	Class teacher	3.34 (72)	41.7	51.4	5.6	1.4
	School social worker	3.33 (53)	41.5	50.9	7.6	0
	School psychologist	3.13 (41)	43.9	34.2	14.6	7.3
	Principal	3.54 (17)	52.9	47.1	0	0
	Guidance counsellor	3.54 (20)	55.0	40.0	5.0	0
	Another teacher	3.53 (20)	55.0	45.0	0	0
*Outside school*	Psychotherapist	3.45 (92)	52.2	41.3	6.5	0
	General Practitioner	3.13 (49)	24.5	63.3	12.2	0
	Psychiatrist	3.29 (55)	41.8	45.5	12.7	0
	Information centre	3.21 (41)	39.0	46.3	12.2	2.4
	Youth welfare office	3.36 (42)	57.1	26.2	11.9	4.8

Percentages relative to all parents rating the helpfulness of the service

#### Access to psychotherapy

Families seeking psychotherapy for their child contacted an average of 5.5 practices (*SD* = 7.2) with a median of 3.0 (*n* = 98). Among those who secured an initial appointment, 43% waited one month and 26% waited two to three months. All data is presented in Table 4. The average waiting time was 11.7 weeks (*SD* = 16.7) with a median of 5.0 weeks (*n* = 80)^2^.

**Table 4:** Waiting times for psychotherapeutic services

	Time until initial appointment (*n* = 93)	Time until start of therapy (*n* = 64)
Up to 4 weeks	43%	23%
5–12 weeks	26%	36%
More than 12 weeks	18%	30%
No comment	13%	11%

Out of all families seeking support (*n* = 255), 25.5% of the children and adolescents began psychotherapy in the year before the survey (*n* = 65), constituting 4.3% of the total sample. Those with higher SDQ scores were more likely to start psychotherapy (Odds ratio = 1.2, *p* < .001). Waiting times between starting to seek help and the start of therapy were longer than for initial appointments, with most families waiting two to three months (36%) or over three months (30%). The average waiting time was 17.9 weeks (*SD* = 20.7), with a median of 10.0 weeks (*n* = 57). See Table 4 for details.

## Discussion

The aim of this study was to assess the current behavioural and emotional problems and quality of life (QoL) in German children and adolescents with an emphasis on predefined risk subgroups. Additionally, we explored the help-seeking behaviour of families of children with mental health issues both within and outside the school context.

While the prevalence of behavioural and emotional problems (21.1%), as well as low QoL (27.3%), was lower than during the pandemic, it had not returned to pre-pandemic levels. Children from low-income families were especially at risk for both behavioural and emotional problems and low QoL. Gender and age differences varied across measurements and data sources.

Approximately one-quarter of parents (23.7%) reported a need for help due to their child’s psychological issues. The majority (70.3%) of families who recognized the need for support pursued it, reaching out both within and outside the school context. Class teachers, psychotherapists and general practitioners were the most contacted services. Although 90.6% of parents who sought help were successful, they often had to contact multiple services to do so. Overall parents reported high satisfaction with accessed psychosocial services in and outside of schools. However, waiting times for therapy were 11.7 weeks for the initial appointment and 17.9 weeks for the start of therapy, which does not align with recommendations for timely interventions.

The prevalence of behavioural and emotional problems in German children and adolescents has changed multiple times over the last six years according to multiple data sources. Prior to the COVID-19 pandemic, the rate was 17.6%, increasing to 30.9% during the height of the pandemic [44]. By autumn 2022, the rate had decreased to 22.6%, closely aligning with our findings of 21.1% [5]. Overall, this trend suggests that mental health issues have stabilised at levels above pre-pandemic figures. Similarly, the proportion of children and adolescents reporting low QoL in this study (27.3%) is higher than pre-pandemic levels (15.3%) but lower than during the height of the pandemic (47.7%) [8, 26]. The prevalence of low QoL has remained relatively stable since autumn 2022 (27.0%), indicating a corresponding stagnation above pre-pandemic rates [5]. A recent unpublished study from the COPSY project (impact of COVID-19 on psychological health) from autumn 2024 found similar trends in their survey of German parents [45].

Several factors may explain why the prevalence of behavioural and emotional problems, as well as low QoL, has not returned to pre-pandemic levels. First, during the pandemic, the rise in mental health issues put additional strain on already insufficient treatment capacities [23, 32]. As long-term mental illness in adolescents bears the risk of chronicity, untreated cases and those not undergoing spontaneous remission may still persist [46]. Second, children and adolescents are facing new crises. For example, a recent study found a link between pandemic-, war- and climate-related distress and poor mental health in secondary school students in Germany [14]. In particular, the phenomenon of eco-anxiety, the intense negative affect triggered by climate-change concerns, has been identified as a salient risk factor for children and adolescents [47, 48]. Third, bullying, including the recent rise in cyberbullying among German children and adolescents, is negatively associated with QoL and other mental health markers, potentially influencing the present cohort [49–51]. Fourth, patterns of social media engagement constitute an additional explanatory variable. Both excessive use and complete abstention are related to poorer wellbeing in adolescents [52]. As problematic social media use in this age group has increased during the pandemic and remained elevated since, this could have influenced results, although effects in studies are typically small [53, 54]. Finally, the rates of child poverty have remained high in Germany and even increased prior to 2024 [55, 56]. The robust association between poverty and adverse mental health outcomes suggests that socioeconomic hardship may partially underlie the sustained prevalence of psychological problems [57]. Further research is needed to determine the factors driving the elevated prevalence of mental health issues.

Our findings, that participants from lower-income families and, in parental report, with lower parental education reported more behavioural and emotional problems and lower QoL align with previous research on the impact of SES on mental health during the pandemic [4, 19]. Low-SES families faced increased risks for stressors such as financial hardship, food and housing insecurity, and unemployment, which negatively affected their psychological well-being [58, 59]. The accumulation of stressors is one of the mechanisms through which low SES adversely affects mental health [17]. Therefore, the long-term effects of pandemic-related stressors likely contributed to the elevated mental health issues observed in this population. Preventing mental health issues thus requires addressing childhood poverty and improving the socioeconomic conditions of children and adolescents. Approximately one-quarter of German children and adolescents currently live in households reliant on governmental assistance [60]. Until this socioeconomic disparity is remedied, a persistently high prevalence of mental health issues is likely.

Regarding age and gender differences, our results were inconsistent across measurements and data sources. In self-reports, adolescent boys reported the lowest SDQ scores, indicating fewer behavioural and emotional problems, while in parental reports, pre-adolescent boys had the highest scores, indicating more problems. This discrepancy may reflect adolescents’ tendency to underreport and parents’ inclination to overreport externalising symptoms [26, 27]. Regarding QoL, adolescent girls reported significantly lower KIDSCREEN-10 scores, indicating lower quality of life, which is consistent with prior findings [8]. A meta-analysis of pandemic-related gender differences found mixed evidence, although more studies identified female gender as a risk factor. Evidence suggests that effects on genders varied, with female adolescents being more susceptible to internal symptoms and stress, while male adolescents faced higher risks of attention problems and addictive gameplay [4]. These findings point to the need for gender-specific interventions.

Additionally, the study revealed new evidence on families’ perceived need, help-seeking behaviour and waiting times for psychotherapy, which have implications for resourcing. Of the parents in our study, 23.7% reported a perceived need for psychosocial help due to their child’s mental health issues, a figure similar to the 20 to 21% reported during the pandemic [61]. The strong association between abnormal or borderline SDQ scores as well as low KIDSCREEN-10 scores and the parent-reported need for help indicates that symptom burden is associated with greater perceived need for support, supporting the validity of the measures. Of parents who expressed a perceived need for support, about a quarter did not seek it, possibly due to common barriers, such as a lack of knowledge on mental health resources, self-stigmatisation, public stigma, logistical barriers and a shortage of professional support [62, 63]. Notably, 23.5% of parents seeking support within and 12.9% outside the school context were unable to find it there, though the reasons for this remain unclear. Class teachers and general practitioners were among the most frequently accessed services, underscoring their crucial role as referral points for professional support as demonstrated in previous studies [33, 64]. As shown in other research, school-based support services are not sufficient [65]. The results indicate a pressing need to expand mental-health services, particularly low-threshold interventions within schools and primary-care settings. A particular emphasis should be placed on primary prevention, aiming to prevent the emergence of disorders before their onset rather than addressing symptoms after they have worsened. In order to mitigate the high prevalence of mental health issues, primary prevention strategies should be implemented in settings that are routinely accessed by young people, such as schools [21]. Although some post-pandemic pilot initiatives have been implemented, e.g., school-based mental health coaches, no nationwide programmes have been established to systematically address youth mental health. The current German government has identified child and adolescent mental health as a policy priority and pledged to develop a “mental health for young people” strategy. To date, no nationwide measures have been implemented, underscoring the necessity for ongoing surveillance.

As the first line of treatment for most mental disorders, psychotherapy is an especially important mental health service, that, nevertheless, remains a limited resource, often unavailable to children and adolescents in need even in high-income countries such as Germany [23, 66]. Our results indicate that children and adolescents with elevated SDQ scores are more likely to initiate psychotherapy, suggesting that higher symptom burden is associated with increased likelihood of treatment uptake. Our findings that, after the end of the COVID-19 pandemic, families had to wait an average of 12 weeks for an initial consultation and 18 weeks for the start of therapy, suggest that waiting times for psychotherapy are still immoderate. Additionally, it should be noted that these waiting times only reflect those children and adolescents who successfully received access to outpatient psychotherapy and might therefore underestimate average waiting times in the population. Furthermore, as acknowledged in the limitations, the restricted sample and potentially unstable estimates necessitate cautious interpretation. Future research should examine help-seeking behaviour and therapy wait times using larger, more robust samples.

In Germany, the statutory health insurance system operates a centralised appointment service (“Terminservicestellen”) that is mandated to arrange an initial psychotherapy consultation within four weeks and within two weeks for urgent cases [67]. Empirical data, including the present study, indicates that these time-frame requirements are routinely unmet, most likely owing to insufficient service capacity [68].

These extended waiting times could have implications for health outcomes. Given the developmental sensitivity of childhood and adolescence, timely treatment of mental disorders is crucial to prevent chronicity and comorbidities and to reduce lifetime disease burden [2, 46]. Extended waiting times compromise these goals and may increase the risk of chronic mental health problems and erode trust in the mental health system [23]. In the long term, childhood mental illness predicts adult unemployment, increasing reliance on state benefits [69]. In 2023 the total cost of mental illness in Germany reached €63.3 billion, imposing substantial pressure on statutory health insurance budgets [70]. Consequently, timely intervention for youth mental health constitutes a cost-effective public-health strategy.

The study has several limitations. As data was collected exclusively in German, the results are not applicable to communities with migration backgrounds who are not fluent in German. Additionally, the sample of participants with low socioeconomic status was small, which limits the conclusions we can draw about the impact of low SES on mental health. To analyse predefined risk subgroups, subgroup analyses were conducted, reducing sample size and increasing the burden of multiple testing. This design limits the depth of conclusions regarding risk populations, especially those excluded, and the underlying mechanisms of vulnerability. The cross-sectional nature of the data prevents causal inferences and the analysis of help-seeking behaviour was based on a relatively small sample and affected by missing data and outliers. Finally, the subscale “conduct problems” exhibited insufficient internal consistencies, permitting only tentative interpretation.

The findings of this study have several practical implications. Class teachers and general practitioners often serve as the first points of contact for families seeking support and should receive appropriate resources and training. Clear referral guidelines and sufficient time allocation for these tasks are essential. Additionally, psychotherapeutic care capacities funded by statutory health insurance should be adjusted based on scientific demand planning to cover increased demand and reduce waiting times [71]. Finally, more preventative measures within school are needed to promote help-seeking behaviour among young people.

## Conclusions

The prevalence of behavioural and emotional problems in this study was 21–22% and the rate of low QoL was 28%, indicating a stabilisation at rates higher than the pre-pandemic baseline. Children from low-income families were particularly at risk. When parents sought support, it was generally rated as helpful, though waiting times for therapy were 12 weeks for the initial appointment and 18 weeks for the start of therapy, which does not align with recommendations for timely interventions.

Findings indicate a need for an expansion of mental health care structures, with a particular focus on schools, as they are often families’ first point of contact. A planned follow-up study will employ a longitudinal design to monitor the development of behavioural and emotional problems and low QoL, while identifying potential intervention strategies.

## Supplementary Information

Additional File 1. German School Barometer Survey (Items in the expert-developed survey relevant to the article translated into English).

## Data Availability

Data is available from the authors upon reasonable request and with permission of the Robert Bosch Foundation.

## Footnotes

1 To measure agreement in SDQ categorisation between parents and their children, Spearman’s rank correlations coefficient was calculated. It showed a high agreement with Spearman’s *ρ* = 0.51. Rank correlation between subscale categorisation was similar (emotional symptoms Spearman’s *ρ* = 0.48, conduct problems *ρ* = 0.41, hyperactivity *ρ* = 0.46 and peer problems *ρ* = 0.54).

2 Extreme values were excluded due to their implausibility.

## Abbreviations

ANOVA: Analysis of variance
CASMIN: Comparative analysis of social mobility in industrial nations
CI: Confidence interval
COPSY: Impact of COVID-19 on psychological health
COVID-19: Coronavirus disease 2019
QoL: Quality of life
KIDSCREEN-10: Kidscreen-10 Index
SDQ: Strength and Difficulties Questionnaire
SES: Socioeconomic status
SHI: Statutory health insurance
Tukey’s HSD: Tukey’s honest significance test
