# Supplementary material for: Mental health, quality of life and mental health service use after the COVID-19 pandemic: Results from a cross-sectional nationwide study among German children and adolescents
Source: BMC Public Health. 2026 Jun 19;26:2115. doi: 10.1186/s12889-026-28159-6 (PMC13352875; doi:10.1186/s12889-026-28159-6)
Supplement: Supplementary file 1 — Additional File 1. German School Barometer Survey (Items in the expert-developed survey relevant to the article translated into English). [file 12889_2026_28159_MOESM1_ESM.docx]

**Additional File 1: German School Barometer Survey**

Only questions relevant to the article “Mental health, quality of life and mental health service us after the COVID-19 pandemic: Results from a cross-sectional nationwide study among German children and adolescents” have been included.

**Screening**

1. Are there any children or adolescents aged between 8 and 17 years living in your household for whom you have custody?

- yes, one child
- yes, several children
- no

IF „YES, SEVERAL CHILDREN“ ON QUESTION 1

1a) How many children or adolescents aged between 8 and 17 years live in your household for whom you have custody?

- NUMERICAL ANSWER
- not applicable

IF “YES, SEVERAL CHILDREN” ON QUESTION 1
For statistical reasons, the following questions only refer to the child aged between 8 and 17 years who had their birthday most recently. Please answer all questions with reference to this child only.

1. How old is this child?

- NUMERICAL ANSWER
- don’t know/not applicable

1. What is your child’s gender?

- male
- female
- diverse
- not applicable

**Questions about mental issues of the child and psychosocial support services**

1. Do you think your child has needed or could have needed help due to mental health issues in the last 12 months?

- yes
- no
- don’t know/not applicable

IF „YES“ ON QUESTION 4

1. In the last 12 months, have you tried to get help for your child inside or outside school due to mental health issues of your child?

- yes
- no
- don’t know/not applicable

IF „YES“ ON QUESTION 5

1. Have you tried to get professional help for your child **at school** in the last 12 months?

(multiple answers possible)

- no
- yes, with a guidance counsellor
- yes, with a school social worker
- yes, with a school psychologist
- yes, with a class teacher
- yes, with another teacher
- yes, with the principal
- yes, with someone else: TEXT FIELD
- don’t know/not applicable

IF „YES“ ON QUESTION 6

1. Who at the school has provided professional help for your child in the last 12 months?

(multiple answers possible)

- guidance counsellor
- school social worker
- school psychologist
- class teacher
- another teacher
- principal
- others
- no help received
- don’t know/not applicable

IF “GUIDANCE COUNSELLOR” ON QUESTION 7

1. a) How helpful was the professional help you received for your child from the guidance counsellor?

- not helpful at all
- not very helpful
- somewhat helpful
- very helpful
- don’t know/not applicable

IF “SCHOOL SOCIAL WORKER” ON QUESTION 7

8. b) How helpful was the professional help you received for your child from the school social worker?

- not helpful at all
- not very helpful
- somewhat helpful
- very helpful
- don’t know/not applicable

IF “SCHOOL PSYCHOLOGIS” ON QUESTION 7

1. c) How helpful was the professional help you received for your child from the school psychologist?

- not helpful at all
- not very helpful
- somewhat helpful
- very helpful
- don’t know/not applicable

IF “CLASS TEACHER” ON QUESTION 7

8. d) How helpful was the professional help you received for your child from the class teacher?

- not helpful at all
- not very helpful
- somewhat helpful
- very helpful
- don’t know/not applicable

IF “ANOTHER TEACHER” ON QUESTION 7

8. e) How helpful was the professional help you received for your child from the other teacher?

- not helpful at all
- not very helpful
- somewhat helpful
- very helpful
- don’t know/not applicable

IF “PRINCIPAL” ON QUESTION 7

8. f) How helpful was the professional help you received for your child from the principal?

- not helpful at all
- not very helpful
- somewhat helpful
- very helpful
- don’t know/not applicable

IF “YES” ON QUESTION 5

1. And have you tried to get professional help for your child somewhere **outside of school** in the last 12 months?

(multiple answers possible)

- no
- yes, with an information centre
- yes, with a psychotherapist
- yes, with a psychiatrist
- yes, with a general practitioner
- yes, with the youth welfare office
- yes, somewhere else: TEXT FIELD
- don’t know/not applicable

IF “YES” ON QUESTION 9

1. Who provided your child with professional help?

(multiple answers possible)

- information centre
- psychotherapist
- psychiatrist
- general practitioner
- youth welfare office
- others: TEXT FIELD
- no help received
- don’t know/not applicable

IF “ INFORMATION CENTRE” ON QUESTION 10

11. a) How helpful was or is the professional help your child received from the information centre?

- not helpful at all
- not very helpful
- somewhat helpful
- very helpful
- don’t know/not applicable

IF “PSYCHOTHERAPIST” ON QUESTION 10

1. b) How helpful was or is the professional help your child received from the psychotherapist?

- not helpful at all
- not very helpful
- somewhat helpful
- very helpful
- don’t know/not applicable

IF “PSYCHIATRIST” ON QUESTION 10

11. c) How helpful was or is the professional help your child received from the psychiatrist?

- not helpful at all
- not very helpful
- somewhat helpful
- very helpful
- don’t know/not applicable

IF “GENERAL PRACTITIONER” ON QUESTION 10

11. d) How helpful was or is the professional help your child received from the general practitioner?

- not helpful at all
- not very helpful
- somewhat helpful
- very helpful
- don’t know/not applicable

IF “YOUTH WELFARE OFFICE” ON QUESTION 10

11. e) How helpful was or is the professional help your child received from the youth welfare office?

- not helpful at all
- not very helpful
- somewhat helpful
- very helpful
- don’t know/not applicable

IF “YES, WITH A PSYCHOTHERAPIST” ON QUESTION 9

1. How many different psychotherapy practices did you try to get an appointment for an initial consultation (e.g. by calling the practice)?

- NUMERICAL ANSWER
- don’t know/not applicable

IF “PSYCHOTHERAPIST” ON QUESTION 10

1. How much time passed between the start of the search for help and the initial consultation with a psychotherapist?

- NUMERICAL ANSWER

____ weeks

- don’t know/not applicable

IF “PSYCHOTHERAPIST” ON QUESTION 10

1. Have you started regular psychotherapy with your child in the last 12 months that is covered by your health insurance?

- yes
- no
- don’t know/not applicable

IF „YES“ ON QUESTION 14

1. How much time passed between the start of the search for help and the start of regular psychotherapy in a psychotherapy practice?

- NUMERICAL ANSWER

_____ weeks

- don’t know/not applicable

**Sociodemographic information about parents/household**

1. Was one of the child’s parents born outside Germany?

- no, both parents were born in Germany
- yes, the mother was born outside Germany
- yes, the father was born outside Germany
- yes, both parents were born outside Germany
- don’t know/not applicable

1. Was your child born in Germany?

- yes
- no
- don’t know/not applicable

1. Is there a person with whom you share custody of the child?

- yes, the person is living with me in the household

- yes, the person is NOT living with me in the household

- no

- not applicable

1. How many people live in your household permanently, including yourself? Please also include all children living in the household.

- NUMERICAL ANSWER

____ people

1. How many children below the age of 14 live in your household?

- NUMERICAL ANSWER
- not applicable

1. What is your household’s total monthly **net** income?

This refers to the total amount of wages, salaries, income from self-employment, pensions and retirement benefits **after deduction of taxes and social security contributions**. Please also include income from public subsidies, income from renting or leasing property, housing benefits, child benefits and other income.

- NUMERICAL ANSWER
- don’t know/not applicable

1. What is your highest school qualification?

- did not attend school
- left school without a qualification
- qualification from a special needs school
- graduated from polytechnic secondary school after 8th grade
- qualification from a secondary school after 8th or 9th grade
- intermediate school leaving certificate/secondary school leaving certificate/graduated from polytechnic secondary school after 10th grade (intermediate qualification)
- basic vocational training year/vocational school/vocational college
- university entrance qualification/technical college entrance qualification/A-levels
- other qualification
- don’t know/not applicable

1. And what professional or academic training do you have?

(multiple answers possible)

- no completed training
- completed apprenticeship, commercial school diploma
- qualification from a specialised secondary school/vocational school/vocational college/technical secondary school (or a comparable qualification from abroad)
- graduated from a professional school/master craftsman or technician school/health care school/graduated from a vocational academy/technical academy (or a comparable qualification from abroad)
- technical college degree/diploma
- university degree (bachelor’s/master’s or equivalents)
- doctorate (doctoral examination)
- other professional qualification (e.g. abroad)
- don’t know/not applicable

IF “YES” ON QUESTION 18

1. What is the highest school qualification of the person with whom you share custody of the child?

- did not attend school
- left school without a qualification
- qualification from a special needs school
- graduated from polytechnic secondary school after 8th grade
- qualification from a secondary school after 8th or 9th grade
- intermediate school leaving certificate/secondary school leaving certificate/graduated from polytechnic secondary school after 10th grade (intermediate qualification)
- basic vocational training year/vocational school/vocational college
- university entrance qualification/technical college entrance qualification/A-levels
- other qualification
- don’t know/not applicable

IF “YES” ON QUESTION 18

1. And what professional or academic training does the person with whom you share custody of the child have?

(multiple answers possible)

- no completed training
- completed apprenticeship, commercial school diploma
- qualification from a specialised secondary school/vocational school/vocational college/technical secondary school (or a comparable qualification from abroad)
- graduated from a professional school/master craftsman or technician school/health care school/graduated from a vocational academy/technical academy (or a comparable qualification from abroad)
- technical college degree/diploma
- university degree (bachelor’s/master’s or equivalents)
- doctorate (doctoral examination)
- other professional qualification (e.g. abroad)
- don’t know/not applicable
